# Healthy enough to work up to age 67 and beyond? A longitudinal population-based study on time trends in working life expectancy free of cardiovascular diseases based on German health insurance data

**DOI:** 10.1136/bmjph-2023-000400

**Published:** 2024-01-04

**Authors:** Jelena Epping, Fabian Tetzlaff, Lieselotte Mond, Juliane Tetzlaff

**Affiliations:** 1 Medical Sociology Unit, Hannover Medical School, Hannover, Germany; 2 Division of Social Determinants of Health, Robert Koch Institut, Berlin, Germany

**Keywords:** Cardiovascular Diseases, Epidemiology, trends

## Abstract

**Background:**

Due to the rising life expectancy in Western countries, the discussion about the increase in the retirement age is becoming more important. However, the prolongation of working lives cannot be implemented successfully without considering the health status of the (potential) employees. In this study, time trends in working life expectancy (WLE) free of widespread cardiovascular diseases (CVD) as well as the proportion of CVD-free working life years are reported.

**Methods:**

Claims data from a German statutory health insurance provider is used to analyse CVD-free WLE. Three periods were defined to assess time trends: 2006–2008 (n=2 075 248), 2011–2013 (n=2 302 127) and 2016–2018 (n=2 579 985). Based on transition rates between the states labour force, non-labour force, CVD and death, CVD-free years spent in the labour force were estimated for each age using multistate life table analyses.

**Results:**

The length of CVD-free WLE increased over time. This increase was stronger in women than in men (+6.4 years vs +2.4 years at age 18). Given the increase in total WLE of the study population, the proportion of CVD-free WLE in total WLE remained stable over time.

**Conclusions:**

The results show that working life years free of CVD increased strongly over the last 13 years and can keep pace with the increase in the length of working lives. Healthier working conditions as well as more efforts in promoting healthy nutrition and less sedentary behaviour could help to further reduce the incidence of CVD and thus contribute to longer healthy working lives.

**WHAT IS ALREADY KNOWN ON THIS TOPIC**

- Due to the rising retirement age, working life expectancy (WLE) increases over time. It remains unclear if this extension of working lives is achieved by working longer in poor or in good health. Previous studies considered general indicators like self-rated health or long-standing illness. This study focuses on cardiovascular diseases (CVD), which are one of the main reasons for labour market exit, and enables thus a discussion about specific prevention measures.

**WHAT THIS STUDY ADDS**

- This study adds evidence on the development of cardiovascular health among labour force over time, showing marked increases of working life years free of CVD for men and even more pronounced for women. Against the background of increasing WLE, the proportion of CVD-free WLE remained stable over time.

**HOW THIS STUDY MIGHT AFFECT RESEARCH, PRACTICE OR POLICY**

- Since the CVD-incidence rates stagnated in more recent years between 2011–2013 and 2016–2018, it is uncertain whether the positive development of CVD-free WLE will continue in the future, especially against the backdrop of the current discussion on further increases of the retirement age. Improved working conditions regarding stress and sitting behaviour could help reduce the risk of CVD and extend the proportion of CVD-free WLE in the future.

## Introduction

 Rising life expectancy in Germany and other Western countries forces social security systems to adapt the regulations regarding statutory pensions. As a result, in many countries, the retirement age has been raised in order to maintain the financing of statutory pension funds.^1 2^ However, the extension of working lives cannot be implemented successfully without considering the health status of the labour force, especially of older persons. One of the main reasons for leaving the labour market due to health reasons are cardiovascular diseases (CVD).^3 4^ For Germany, there is still insufficient data on the length of life free of specific diseases in the working-age population, which could help to implement specific prevention measures. This study focuses on widespread CVD and examines for the first time the development of working life years free of CVD over time.

Working life expectancy (WLE) is an established measure to capture the length of working lives.^5^ Eurostat uses WLE for comparisons of working life duration between countries as well as over time.^6^ The common result of these studies is an increase in WLE over time, though differing between social groups^7 8^ or countries.^9 10^ While extending working lives reduces the pressure on pension systems, it remains unclear whether this policy goal is achieved by working longer in poor health if the health of the working-age population is not considered.^11^

In 2007, Lievre *et al* introduced healthy working life expectancies (HWLE) as an indicator that combines information on labour force participation and health.^12^ Based on the data from the European Community Household Panel, the authors showed that men spend about half of the years they live in good health working; for women the proportion was about one-third.^12^ The findings suggested that a reservoir of healthy years existed that could be spend working.^12^

Numerous studies examined WLE with regard to disability or long-standing illness,^13 14^ chronic diseases^15 16^ or specifically osteoarthritis.^17^ To our best knowledge, there are no studies on WLE with respect to CVD up to now. The best approximation was the study on exit from paid employment, which found a twofold risk of exit from paid employment due to disability benefits for persons with CVD compared with persons without CVD.^18^ Overall, a systematic review analysing several population indicators of health and work concluded that HWLE is a suitable measure for monitoring life expectancy in health and work that is still rarely used in research.^19^ Furthermore, only a few studies analysed the development of WLE free of limitations or diseases over time. In Finnish^14^ and German^20^ studies, based on survey data, an increase in HWLE (based on subjective health indicators) could be shown between 2000 and 2018. An overview for 14 countries reported an increase in HWLE as well, except for men in the USA.^21^

Most of the studies on WLE and HWLE were performed based on survey data: English Longitudinal Study of Aging,^17 22^ Longitudinal Aging Study Amsterdam^15^ or Dutch Study on Transitions in Employment, Ability and Motivation.^18 23^ Only a few studies, mostly from Scandinavian countries, used register data (eg,^13 24^ most probably due to strict regulations on data protection in many countries including Germany). For Germany, health insurance data provide a suitable database to analyse (H)WLE and were recently used in a first study to calculate WLE.^25^ This study revealed that WLE in Germany increases over time at every age up to the highest working age,^25^ which reflects well the trend observed in previous studies based on survey data.^7 20^ Compared with survey data, routinely collected data from population registers or health insurance providers have several advantages, for example, the detailed information on morbidity (diagnoses, medication), employment status and mortality. The longitudinal nature of the data and the high case numbers allow for analysing time trends in HWLE with respect to specific diagnoses.

The aim of this innovative study was to investigate trends in WLE free of CVD in men and women between 2006 and 2018 based on German statutory health insurance (SHI) data, previously used for the calculation of WLE.^25^ The study is guided by the following questions:

1. How did the WLE free of CVD develop over time?
2. Are there differences between men and women in CVD-free WLE?
3. How did the proportion of working years free of CVD in total working years (healthy working ratio) develop over time?

## Methods

The analyses were based on the insurance population of the German SHI provider AOK Niedersachsen covering about 37% of the inhabitants of Lower Saxony.^26^ Membership of health insurance is mandatory in Germany. Above a certain threshold (gross income about 36 Tsd € in 2019), it is possible to choose between private and SHI. Due to the high income threshold, about 89% of the German population are insured within SHI system.^27^ After the payment of the mandatory insurance fee, the provision of health services is free of charge covering a wide range of health services. Civil servants and self-employed persons are usually not members of SHI, since other conditions apply due to legal regulations.

The data contain sociodemographic variables (sex, birth year) as well as dated information on insurance entry and exit, and on employment status. The employment status is recorded within the SHI data since, according to the legal regulations, the insurance fee for employed persons is transferred from the employer directly to the SHI as part of the social security payment. Therefore, the exact date of the start and end of the employment or unemployment are known to the SHI and can be used in the analyses of WLE.

Information on diseases is obtained from outpatient and inpatient diagnosis data (ICD-10 GM). Physicians are obliged to register medical treatment cases, including the corresponding diagnoses to get the refund of their services from an SHI provider; the same applies for hospitals.

### Cardiovascular diseases

Several diseases were combined into the indicator CVD: chronic heart disease, heart failure, angina pectoris and peripheral artery disease as chronic diseases, and myocardial infarction and stroke as major cardiovascular events (online supplemental table S1 for ICD-10 codes). The following case definition criteria were applied:

1. If a diagnosis appeared only in outpatient data, a confirmation through a repeated diagnosis in another quarter of the same year was obligatory.
2. Diagnoses documented in hospitals that were not the main cause of treatment had to be confirmed by a repeated diagnosis in the same year.
3. If a diagnosis documented the main cause of inpatient treatment, the case did not require a confirmation.

### Time trends and definition of incident cases

Time trends were assessed by splitting the available data into three periods: 2006–2008, 2011–2013 and 2016–2018. For the definition of incident CVD cases, a preobservation period of 365 disease-free days was used, which depicts the usual procedure for SHI data.^28^ The monitoring of CVD is organised in Germany quite well within outpatient healthcare for chronic diseases, especially due to regular visits for prescribing medication, and inpatient care for cardiovascular events. Thus, it is unlikely that a prevalent case would not be detected within 365 pre-observation period.

Insured persons were considered as incident cases only if the diagnosis was assigned at least 365 days after their individual insurance period started to ensure sufficient time of preobservation. Individuals having a CVD diagnosis at the beginning of their individual preobservation period were considered prevalent and were excluded from the analyses. The share of excluded individuals was low (ca. 2.5%) and remained stable over time.

### CVD-free WLE

For the calculation of WLE, the labour force concept of the International Labour Organization was used.^29^ Accordingly, employed and self-employed persons as well as unemployed persons were assigned to the labour force. If a person left the labour market and changed the insurance status, for example, to pensioner, the status non-labour force was assigned. Using the dated information on status change, the exact period of belonging to the labour force was defined on an individual basis. To cover a broad range of working age, persons aged 18–69 years were considered. More detailed information on the methodological issues and the results on time trends in WLE of the same study population can be found in a previous study.^25^

**Figure 1:**
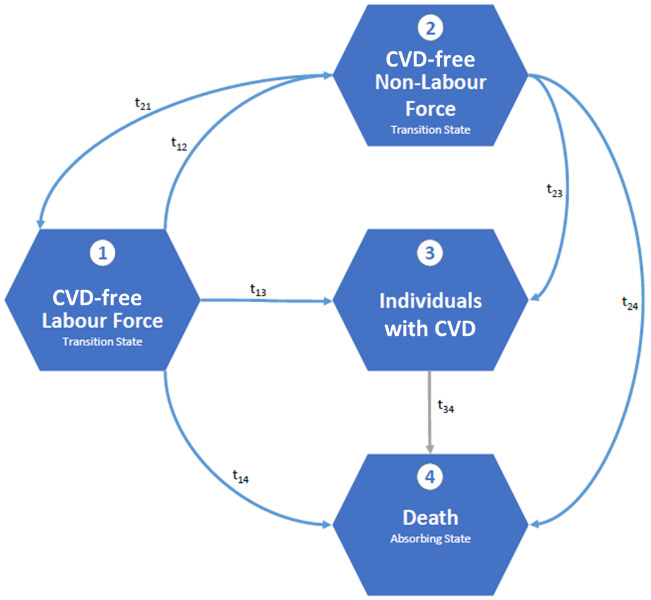
Transition modelling between non-labour force, labour force, CVD prevalence and death. CVD, cardiovascular disease.

The information on the working status was then combined with the information on CVD incidence to calculate age-specific transition rates between the four states (figure 1). For example, a person could start in CVD-free non-labour force (eg, still in education), then make the transition to the CVD-free labour force (t_21_) before he or she gets incident with CVD (t_13_) or dies (t_14_). The transitions between labour force and non-labour force can occur several times within the observation period.

Based on these transition rates, CVD-free years spent in the labour force were estimated for each age using multistate life table analysis. The analyses were performed stratified by sex and time period using Stata V.16.0 MP and R V.4.2.1. Due to the high case numbers, 95% CIs were very small and the changes in CVD-free WLE were significant up to the highest working age. We, therefore, refrain from reporting them in the line plot. As an example, we report 95% CIs of CVD-free WLE for age 18 and 50 based on 1000 bootstraps.

To assess the relative change in CVD-free WLE over time, the proportion of CVD-free WLE in total WLE (healthy working ratio) was calculated. For this purpose, previously published results on the total WLE based on the same data set were used.^25^ The healthy working ratio allows for comparisons of the level of disease-free WLE between different periods considering the development of WLE over time.

The study was conducted according to the RECORD statement. The calculations were made in Stata MP 16.0.

### Patient and public involvement

No patients were involved. The study is based on pre-existing routinely collected data of the insured persons of a SHI in Lower Saxony, Germany. The data are used in anonymised form. According to the Germany Law (Sozialgesetzbuch V), no patient consent is required for this type of data.

## Results

Table 1 shows the descriptive statistics of the insurance population of the AOK Lower Saxony for the age group 18 to 69 years, stratified by sex and time periods. The age-standardised incidence of CVD in the working age population decreased in men over time. For women, after a distinct decrease between 2006–2008 and 2011–2013, a slight increase can be observed between 2011–2013 and 2016–2018. The proportion of labour force increased over time as well, especially true for women.

**Table 1:** Characteristics of the study population by period and sex, age range 18­­–69 years

	Men			Women		
	Period 1 (2006–2008)	Period 2 (2011–2013)	Period 3 (2016–2018)	Period 1 (2006–2008)	Period 2 (2011–2013)	Period 3 (2016–2018)
Person-years at risk	1 460 876	1 765 282	2 071 061	1 042 209	1 287 135	1 674 684
Median age (IQR)	40 (29–49)	40 (29–50)	38 (28–50)	40 (28–49)	40 (28–50)	39 (28–51)
Age-standardised incidence per 10 000 person-years	147.9	136.5	132.7	87.8	77.4	78.9

**Figure 2:**
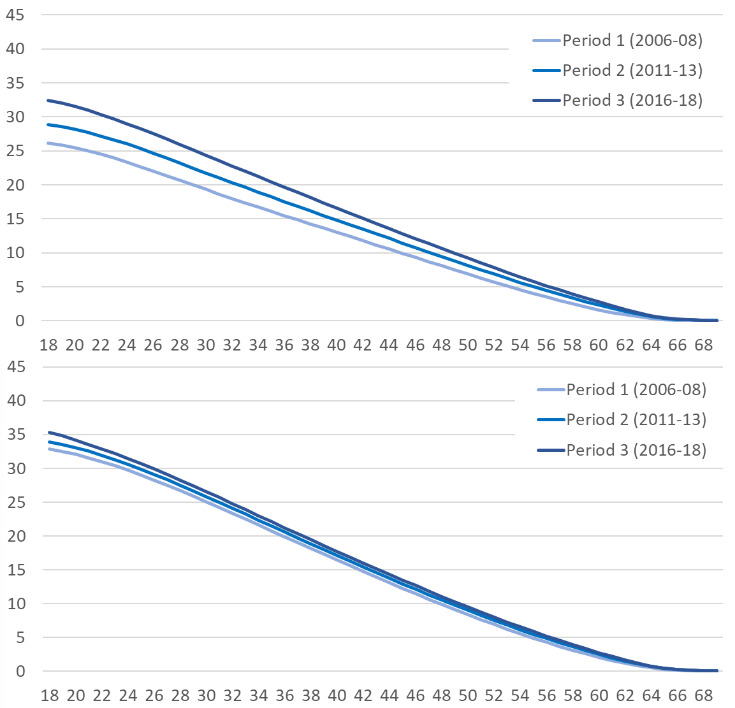
CVD-free working life years by period and sex. The upper panel shows the results for women, the lower panel for men.­ CVD, cardiovascular disease.

CVD-free working life years increased for both sexes over time. As shown in figure 2, this increase was stronger in women than in men, which holds for all considered ages. This led to a decreasing gap in CVD-free WLE between women and men. Despite the narrowing gap between sexes, women have lower levels of CVD-free WLE than men in all three periods.

**Figure 3:**
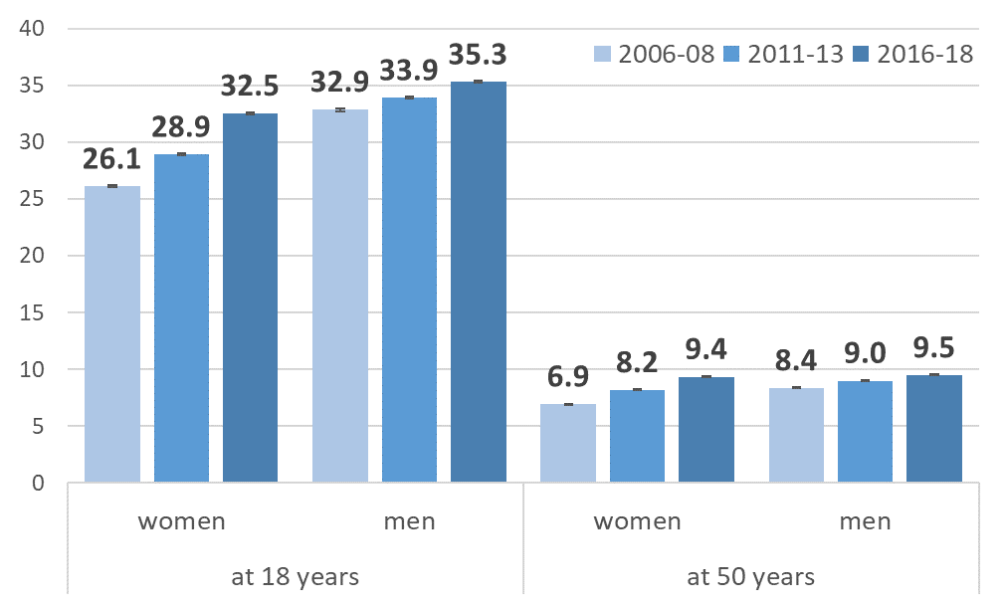
CVD-free working life years for men and women at age 18 and age 50 with 95% CIs. CVD, cardiovascular disease.

Focusing on time trends in CVD-free WLE at age 18 and 50, we observe an increase in CVD-free WLE in women and men aged 18 by 6.4 years and by 2.4 years, respectively (figure 3). For persons at age 50, the increases were also stronger in women than in men (+2.5 years in women and +1.1 years in men). Due to the population size, the CIs are very small, ranging from 0.02 years at the minimum and 0.15 years at the maximum.

**Figure 4:**
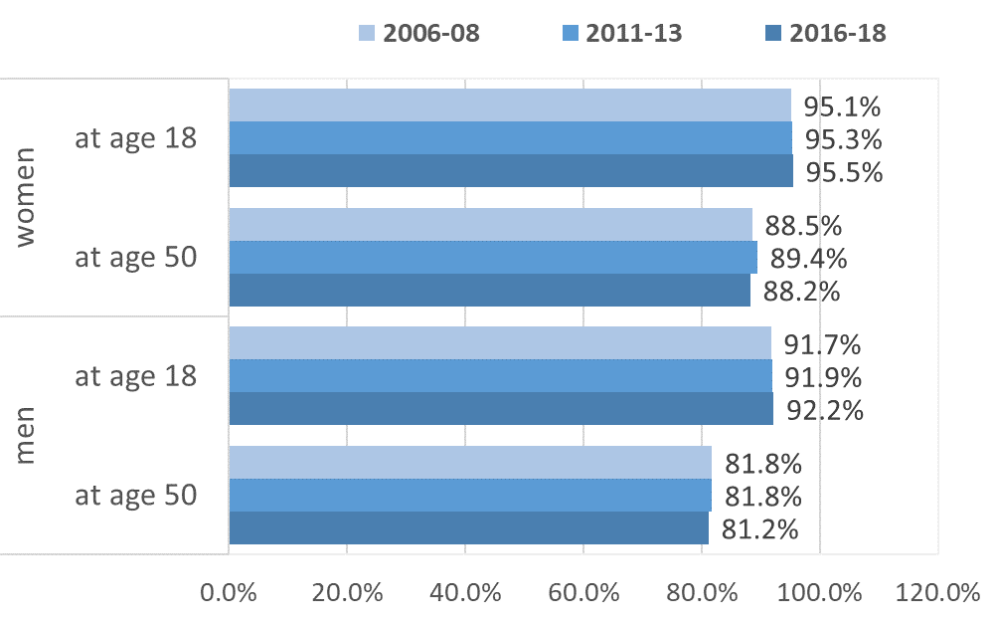
Proportion of CVD-free WLE in total WLE (health ratio) for men and women aged 18 and 50. CVD, cardiovascular disease; WLE, working life expectancy.

Figure 4 presents the proportion of CVD-free WLE in total WLE (healthy working ratio) for men and women at age 18 and 50. Despite the strong increases in CVD-free WLE over time, the healthy working ratio remained largely stable over time.

## Discussion

This study analysed trends in CVD-free WLE based on claims data of a German SHI provider between 2006 and 2018. Our study provides the first evidence on trends in WLE free of CVD in Germany. For the short period of 13 years, substantial increases in CVD-free WLE of up to 6.4 years were found for women. In men, CVD-free WLE was at any age higher than for women. It also increased over time, though at a slower pace meaning that the gap between men and women is decreasing over time. At the same time, the decreases in CVD-incidence rates were more pronounced in men (table 1). The increases in CVD-free WLE should be interpreted against the background of marked prolongation of working lives in men and especially in women between 2006 and 2018.^25^ The proportion of CVD-free WLE in total WLE remained stable in men and women over time, meaning that the increases in CVD-free WLE could keep pace with the increasing working lives. Since the CVD-incidence rates stagnated in more recent years between 2011–2013 and 2016–2018, it is uncertain whether the positive development of CVD-free WLE will continue in the future, especially against the backdrop of the current discussion on further increases of the retirement age.

In concordance with our results, increases in life expectancy free of myocardial infarction or stroke over time could be found in other studies, though it does not hold for all income groups equally.^30 31^ Direct comparisons with other studies on WLE are not possible, since, up to now, there are no studies analysing WLE free of CVD. The analysis of exit from paid employment reported a twofold increased risk for people with CVD compared with those without chronic diseases.^18^

Though CVD-incidence rates in men are higher than in women (table 1), men spent more years in the labour force free of CVD due to higher total WLE in men compared with women.^25^ Such differences between men and women were also found in other studies,for example, analysing limiting long-standing illness,^17 22^ but the evidence on time trends in disease-specific WLE is up to now lacking.

The positive result of increases in healthy WLE should be considered against the background of growing total working life expectancies, reported for the most industrial countries.^7 21^ These increases in total WLE entail that WLE in poor health increased as well. Therefore, relative changes in the proportion of HWLE in total WLE (healthy working ratio) should be considered as well. In our study, the healthy working ratio remained constant over time, indicating that the increase in CVD-free WLE was accompanied by an increase in working years with CVD. This development is most likely induced by the increasing retirement age in Germany and other incentives to work longer, which have led to growing shares of older people staying in the labour market up to higher ages (15% of employed persons aged over 55 years in 2012 grew up to 22% in 2022).^32^ Despite decreases in CVD incidence over time, the strong increase in WLE due to higher labour force participation of older persons has led to more years with CVD spent in the labour market, even though CVD-free WLE increased strongly in parallel. Other studies reported a similar development over time, showing an increase in years with self-reported chronic diseases^21^ in parallel to years without chronic diseases.

The current overall trends in cardiovascular morbidity^33^ and mortality^34^ are quite positive in Germany. However, several processes can influence the future development of the cardiovascular morbidity in the labour force. The ongoing rapid changes in the digitalisation of work and the increasing work density can increase the mental pressure for employees and may contribute to a less favourable trend in cardiovascular morbidity.^35 36^ Furthermore, increased sedentary behaviour at work and in leisure time has a negative influence on the development of the prevalence of such risk factors as diabetes and obesity and, subsequently, on cardiovascular health as well.^37 38^ The increasing labour shortage in Germany, on the other hand, could provide new opportunities for employees to negotiate better working conditions. Schram *et al* showed that a certain share of working years is lost due to unfavourable working conditions.^23^ Better working conditions combined with public efforts on promoting healthy diet and less sedentary behaviour can help to reduce the risks of CVD even further and thus increase the proportion of healthy years in the WLE.

### Strengths and limitations

This study is based on claims data of the SHI provider AOK Lower Saxony, covering about 37% of the inhabitants of the federal state Lower Saxony. The study population is representative of the whole population of Lower Saxony and Germany in terms of sex and age distributions, while persons with lower socioeconomic status are overrepresented.^39^ Since several studies showed a positive association between education and (healthy) WLE,^7 8 23^ the levels of CVD-free WLE may be somewhat underestimated in our study as compared with the entire population of Germany.

The assignment of the diagnosis in claims data is performed for the purpose of claiming refund for medical services. We applied validation criteria during the case selection, though we cannot fully rule out that some persons are not CVD-incident in spite of the repeated documented diagnosis in our data. At the same time, we cannot fully rule out that some persons were falsely excluded from the analysis by not fulfilling the criteria. Due to data availability for a certain time period, the study was performed using a period perspective under the assumption that the conditions of a certain period prevail throughout the lives of the members of this synthetic cohort. The advantage of this approach is depicting the current developments for all ages included in the analyses and thus providing useful insights. Using a real cohort would allow for the description of the development over the whole life course, though current working life conditions for younger individuals at the beginning of their working lives would not be taken into account.

In contrast to survey data, claims data contain individual dated information on the employment status as well as diagnoses, allowing for precise estimation of (CVD-free) WLE. Furthermore, no selection, recall or morbidity bias applies to claims data due to the routine nature of the data and since individuals are included irrespective of their health status. As shown in previous research, the chance to participate in a panel study is lower for persons with physical impairments.^40^ This could lead to lower levels of morbidity in survey data as compared with claims data-based analysis of (H)WLE.

Applying the concept of labour force allows for comparisons over time since economic fluctuations causing increases or decreases in unemployment rates do not influence the analyses. Another strength of the labour force concept is that it includes a broader range of individuals and can thus also reveal potential for the job market.

## Conclusions

To the best of our knowledge, it is the first study analysing WLE free of CVD. Against the background of increasing working lives, this study shows steady increases in working life expectancies free of CVD. Furthermore, the share of CVD-free working years remained constant over time, indicating that the increase in CVD-free work years has kept pace with the increase in WLE but was also accompanied by more years in the labour market with poor cardiovascular health. Better working conditions as well as more efforts in promoting healthy nutrition and less sedentary behaviour in leisure time could help to further reduce the incidence of CVD and thus contribute to longer healthy (working) lives. Further studies should examine if the increases in CVD-free work years apply to all socioeconomic groups.

## supplementary material

10.1136/bmjph-2023-000400 online supplemental file 1

## Data Availability

No data are available.
