# Supplementary material for: Healthy enough to work up to age 67 and beyond? A longitudinal population-based study on time trends in working life expectancy free of cardiovascular diseases based on German health insurance data
Source: BMJ Public Health. 2024 Jan 4;2(1):e000400. doi: 10.1136/bmjph-2023-000400 (PMC11816200; doi:10.1136/bmjph-2023-000400)
Supplement: online supplemental file 1 [file bmjph-2-1-s001.pdf]

Supplement Table S1. Diagnosis codes for the case selection of cardiovascular diseases according to ICD-10-GM (International Classification of Diseases and Related Health Problems, German Modification). Working life expectancy free of cardiovascular diseases, Germany, 2006-2018.

Manuscript titled „ Healthy enough to work up to age 67 and beyond? Working life expectancy free of cardiovascular diseases between 2006 and 2018 in Germany"

I20	Angina Pectoris
I21	Acute myocardial infarction
I22	Subsequent ST elevation (STEMI) or non-ST elevation (NSTEMI) myocardial infarction
I23	Certain current complications following ST elevation (STEMI) and non-ST elevation (NSTEMI) myocardial infarction (within the 28 day period)
I24	Other acute ischemic heart diseases
I25	Chronic ischemic heart disease
I50	Heart failure
I60	Nontraumatic subarachnoid hemorrhage
I61	Nontraumatic intracerebral hemorrhage
I63	Other and unspecified nontraumatic intracranial hemorrhage
I64	Cerebral infarction
I70.2	Atherosclerosis of native arteries of the extremities
I73.9	Peripheral vascular disease, unspecified
